# Genome-Wide Identification of lncRNAs Involved in Fertility Transition in the Photo-Thermosensitive Genic Male Sterile Rice Line Wuxiang S

**DOI:** 10.3389/fpls.2020.580050

**Published:** 2021-01-14

**Authors:** Ying Wang, Hongyuan Zhang, Qian Li, Jing Jin, Hao Chen, Yu Zou, Xingguo Huang, Yi Ding

**Affiliations:** ^1^State Key Laboratory of Hybrid Rice, Department of Genetics, College of Life Sciences, Wuhan University, Wuhan, China; ^2^Wuhan Vegetable Research Institute, Wuhan Academy of Agricultural Science, Wuhan, China; ^3^Wuhan Wuda Tianyuau Bio-Tech Co., Ltd., Wuhan, China

**Keywords:** lncRNA, PTGMS rice, fertility transition, ssRNA-seq, eTMs, dual luciferase reporter assay

## Abstract

Long non-coding RNAs (lncRNAs) act as universal regulators of various biological processes, but no genome-wide screening of lncRNAs involved in the fertility transition of the photo-thermosensitive genic male sterile (PTGMS) rice line has been reported. Here, we performed strand-specific RNA sequencing at three developmental stages of a novel PTGMS line Wuxiang S (WXS). A total of 3,948 lncRNAs were identified; 622 of these were detected as differentially expressed lncRNAs (DE-lncRNAs) between male-sterile WXS (WXS-S) and male-fertile WXS (WXS-F). A large proportion of lncRNAs differentially expressed at the stage of pollen mother cells meiosis, suggested that it may be the most critical stage for fertility transition of WXS. Furthermore, functional annotation of the *cis*- and *trans*- targets of DE-lncRNAs showed that 150 targets corresponding to 141 DE-lncRNAs were identified as involved in anther and pollen development. Moreover, computational analysis predicted 97 lncRNAs as precursors for 72 miRNAs, and 94 DE-lncRNAs as potential endogenous target mimics (eTMs) for 150 miRNAs. Finally, using the dual luciferase reporter assays, we demonstrated that two lncRNAs act as eTMs to regulate the expression of the *SPL* and *GRF* genes by competing for the shared osa-miR156 and osa-miR396, respectively. These genomic characteristics, differential expression, and interaction of lncRNAs with miRNAs and mRNAs contribute to our understanding of the roles of lncRNAs during the fertility transition in PTGMS rice lines.

## Introduction

Rice (*Oryza sativa* L.) is a major food crop, and the development and utilization of hybrid rice in agriculture has contributed significantly to food sufficiency worldwide over the past several decades. On the basis of production technology, hybrid rice can be classified into three-line and two-line systems. The two-line system applies a nuclear-controlled photo-thermosensitive genic male sterile (PTGMS) line that serves not only as a male sterile line but also as a maintainer line (Cheng et al., 2007; Chen and Liu, 2014; Fan and Zhang, 2017). Therefore, the wide application of a two-line system can greatly simplify and significantly improve the efficiency of hybrid rice breeding and hybrid seed production. In general, the male sterility of the PTGMS line, which is characterized by indehiscent anthers, aborted pollen development and abnormal male gametes, is regulated by the interaction of environmental fluctuation with complex genetic and epigenetic elements (Shi et al., 2009; Chang et al., 2016).

In recent decades, several PTGMS gene loci have been identified. For instance, the *Pms1* and *Pms3* loci were shown to control fertility segregation in two crosses between Non-gken58S (NK58S) and other rice varieties (Mei et al., 1999). Interestingly, *Pms1* and *Pms3* both encode long non-coding RNA (lncRNA). *Pms1* encodes PMSIT, a lncRNA that is targeted and triggered by miR2118 to produce 21-nt phased small-interfering RNAs (phasiRNAs). A single nucleotide polymorphism (SNP) near the cleavage site in PMS1T may increase the accumulation of phasiRNAs, causing male sterility of NK58S under long-day conditions (Fan et al., 2016). Similarly, *Pms3* encodes LDMAR, a lncRNA whose promoter may generate a 21-nt small interfering RNA (siRNA), that directs DNA methylation in the promoter region. A SNP may increase methylation of the LDMAR promoter, this specifically reduces the expression of LDMAR, causing male sterility of NK58S under long-day conditions (Ding et al., 2012a, b). Simultaneously, another laboratory identified the *p/tms12-1*, which occurs at the same locus as *PMS3*, also encodes a unique lncRNA that produces a small RNA osa-smR5864w and confers the male sterile of NK58S and Peiai 64S (PA64S) under long-day and high-temperature conditions, respectively (Zhou et al., 2012). Taken together, these studies indicate that lncRNAs are important regulators of male sterility in the PTGMS rice line under certain environmental conditions. However, our knowledge of the lncRNAs involved in fertility transition in PTGMS rice is still poor.

Advances in high-throughput deep sequencing technology have revealed that approximately 90% of the eukaryotic genome is actively transcribed, although a considerable proportion of those transcripts are non-coding RNAs (ncRNAs; Wilhelm et al., 2008). These ncRNAs can be grouped into small ncRNAs and lncRNAs according to their length. In general, lncRNAs constitute a family of transcripts that are more than 200 nt in length and possess no protein-coding capacity; they are often transcribed by RNA polymerase II (Pol II) and are always modified by capping, polyadenylation and splicing (Ponting et al., 2009; Wilusz et al., 2009). LncRNAs can be further classified into sense-lncRNAs, antisense-lncRNAs, intronic-lnRNAs, and intergenic lncRNAs (lincRNAs) based on their locations relative to protein-coding genes in the genome. Recent increasing evidence supports the idea that lncRNAs play regulatory roles in gene expression at the transcriptional, post-transcriptional, and epigenetic levels (Mercer et al., 2009; Wilusz et al., 2009). Identification of lncRNAs has been undertaken in several model plant species, such as *Arabidopsis thaliana* (Wang et al., 2014; Zhu et al., 2014), *O. sativa* (Zhang et al., 2014; Xu et al., 2016), *Zea mays* (Wang et al., 2018), *Triticum aestivum* (Cagirici et al., 2017), and *Medicago truncatula* (Wang et al., 2017). To date, more than 200,000 lncRNAs from 45 plant species have been annotated in the Green Non-Coding Database (GreeNC, http://greenc.sciencedesigners.com/wiki/Main_Page). Several plant lncRNAs have been functionally characterized as participating in multiple biological processes such as leaf development, flowering, sexual reproduction, and biotic and abiotic stress responses (Ma et al., 2008; Heo and Sung, 2011; Song et al., 2013; Qin et al., 2017; Liu et al., 2018). However, no genome-wide identification of lncRNAs that may be involved in fertility transition in the PTGMS rice line has been reported so far.

Wuxiang S (WXS) is a PTGMS rice line, developed by our laboratory (variety right number CNA20120607.9). Our previous studies showed that the fertility transition of WXS is controlled by photoperiod and temperature: it was completely male sterile when grown under the high temperatures (≥23.5°C) and long-day (≥14 h) conditions; in contrast, it becomes fertile under the conditions of low temperature (approximately 21°C) and short day length (12 h) during anthers development. Study of cytological observation indicated that abortion starts from the stage of pollen mother cells (PMCs) formation and throughout the reproductive process (Zhang et al., 2016; Wang et al., 2019). Therefore, WXS is an ideal experimental system in which to identify lncRNAs that act during the fertility transition. In the present study, we collected young panicles from male-sterile WXS (WXS-S) and male-fertile WXS (WXS-F) at three developmental stages and used them to construct 18 RNA-seq libraries for strand-specific RNA sequencing (ssRNA-seq). We aim to perform genome-wide identification and profiling of the lncRNAs that are expressed during the fertility transition in WXS rice. This study will increase our understanding of the role of lncRNAs in PTGMS line and will provide clues that will help further elucidate the molecular mechanisms of fertility transition.

## Materials and Methods

### Plant Materials and Growth Conditions

Wuxiang S is a PTGMS rice line that was selected and bred by our laboratory, and obtained the new plant variety right (CNA20120607.9) granted by the Ministry of Agriculture of the People’s Republic of China. The fertility transition of WXS is controlled by photoperiod and temperature: it was completely male sterile when grown under the high temperatures (≥23.5°C) and long-day (≥14 h) conditions; in contrast, it becomes fertile under the conditions of low temperature (approximately 21°C) and short-day length (12 h) during anthers development. The seeds were obtained from State Key Laboratory of Hybrid Rice, College of Life Sciences, Wuhan University, Wuhan, China. Between May and August 2017, WXS rice was planted in the experimental field of Wuhan University (30°54′ N, 114°37′ E), Wuhan, China. During summer in Wuhan, the daily average temperatures are above 24°C, and there is approximately 14 h of light per day, the conditions that can induce WXS male sterility (WXS-S). Moreover, our previous cytological observations of WXS sterile anthers showed that the PMCs were abnormal; they formed aberrant dyads and tetrads during the subsequent meiosis and eventually produced aborted pollen (Zhang et al., 2016; Wang et al., 2019; Jin et al., 2020). In the sterile period, the total number of spikelets in the panicle was 317.0, stigma exposure rate was 100%, and bilateral exposure rate 74%. For other WXS plants, once the panicle length was approximately 1 cm, the plants were transferred from natural conditions to a fully intelligent artificial climate plant incubator, approximately 21°C and 12 h light/12 h dark photoperiod for 3–4 weeks to cause the transition to WXS male fertility (WXS-F). Young panicles of WXS-S and WXS-F were harvested at three different developmental stages: PMCs formation (P2), meiosis (P3), and microspores formation period (P4); these stages are designated SP2, SP3, SP4, FP2, FP3, and FP4, respectively. After collection, all samples were immediately frozen in liquid nitrogen and stored at −80°C.

### RNA Extraction and Strand-Specific RNA Sequencing

To minimize the deviation between parallel samples of WXS-S and WXS-F at the three different stages described above, each sample was collected from multiple independent panicles, and the samples were pooled; each analyzed sample consisted of three biological replicates. Total RNA was extracted from the young panicles of each sample using TRIzol reagent (TaKaRa, Dalian, China) according to the manufacturer’s instructions. After RNA quality confirmation, 1.5 μg RNA from each sample was treated with the Ribo-Zero rRNA Removal Kit (Epicenter, Madison, WI, United States) to remove ribosomal RNA (rRNA). Subsequently, 18 RNA sequencing libraries were constructed using the NEBNextR UltraTM Directional RNA Library Prep Kit for IlluminaR (NEB, United States) according to the manufacturer’s recommendations. Finally, sequencing of the libraries was conducted on the Illumina HiSeq Xten platform at Biomarker Biotechnology Co., Ltd (Beijing, China).

### LncRNA Identification

Clean reads were obtained by removing the adaptor sequences and low-quality reads from raw reads; the reads were then mapped to the rice reference genome MSU-v7.0^1^ using the software HISAT2 (version 2.0.5). The transcriptome was then assembled by StringTie (version 1.3.1; Pertea et al., 2016), and the assembled transcripts were annotated using the gffcompare program. Subsequently, lncRNAs were identified based on the following steps: first, transcripts with the class codes “I,” “x,” “u,” “o,” and “e” were selected; second, the transcripts were filtered to remove single-exon transcripts shorter than 200 nucleotides in length; third, the transcripts with expected the fragments per kilobase of transcript sequence per million base pairs sequenced (FPKM) value greater than 0.1 were selected. The obtained transcripts were further screened using the analytical methods of Coding Potential Calculator (CPC, score < 0), Coding-Non-Coding Index (CNCI, score < 0), Pfam-scan (Pfam, *E*-value < 0.001), and Coding Potential Assessment Tool (CPAT, score < 0) to distinguish protein-coding genes from non-coding genes. Eventually, we highly reliably identified the putative lncRNAs in the PTGMS rice line WXS. The different types of lncRNAs were selected using the cuffcompare.

### Differential Expression Analysis and Quantitative Real-Time PCR Validation

StringTie (version 1.3.1) was used to calculate the FPKM of the lncRNAs and mRNAs in each sample (Pertea et al., 2016). The FPKMs were calculated based on the lengths of the fragments, and read counts were mapped to this fragment. Differential expression analysis between WXS-F and WXS-S at three different developmental stages was performed using the DESeq R package (version 1.10.1). The resulting *P*-values were adjusted using Benjamini and Hochberg’s approach to control the false discovery rate. LncRNAs and with an adjusted *P*-value < 0.05 and absolute values of log_2_(fold change) ≥ 1 found by DESeq were assigned as differentially expressed lncRNAs (DE-lncRNAs).

To validate the expression of the identified lncRNAs, quantitative real-time PCR (qRT-PCR) was performed using the total RNA extracted from the same samples that were used for RNA-seq. Approximately 1 μg of total RNA was reverse-transcribed into first-strand cDNA using a Fermentas RevertAid First Strand cDNA Synthesis Kit (Fermentas, United States). For the qRT-PCR of lncRNA, cDNA templates were reverse-transcribed using random primers. However, cDNA templates were reverse-transcribed using oligo(dT)18 primers for the qRT-PCR of mRNAs. All cDNAs were diluted 10-fold. qRT-PCR was performed using SYBR-green fluorescence with an ABI StepOnePlus Real-Time PCR System. The Reaction conditions as described in the previous study (Zhang et al., 2016). *OsActin1* was used as the endogenous reference gene. Each set of experiments was repeated three times, and the results were calculated using the comparative C_T_ method. The gene-specific primer pairs and internal control primers are listed in Supplementary Table 1.

### Gene Functional Annotation

The functions of target genes of DE-lncRNAs were annotated based on the following databases: Nr (NCBI non-redundant protein sequences); Pfam (Protein family); KOG/COG (Clusters of Orthologous Groups of proteins); Swiss-Prot (A manually annotated and reviewed protein sequence database); KEGG (Kyoto Encyclopedia of Genes and Genomes); and GO (Gene Ontology). GO enrichment analysis of the target genes of the DE-lncRNAs was implemented using the topGO R package (Alexa and Rahnenfuhrer, 2010). For KEGG pathway enrichment analysis, KOBAS software was used to test the statistical enrichment of target genes of the DE-lncRNAs in KEGG pathways (Mao et al., 2005).

### Prediction of miRNA Precursor of lncRNA

The rice miRNA data were obtained from miRBase^2^. To predict lncRNAs as potential miRNA precursors, the sequences of lncRNAs were aligned to those of rice miRNA precursors using BLAST, and lncRNAs that showed alignments of greater than 90% with miRNA precursors were selected.

### Prediction of lncRNAs As Endogenous Target Mimics

The miRNA-target regulatory relationships were predicted by Target Finder. Then, candidate endogenous RNAs (ceRNAs) pairs were screened based on the presence of no fewer than three shared miRNAs between the lncRNA and the mRNA. Finally, the candidate ceRNA pairs were significant co-regulation networks if they met the criterion that the adjusted combined *P*-value was no larger than a threshold of 5% (*P* ≤ 0.05). The ceRNA networks were visualized using Cytoscape (version 3.5.0).

### Rice Protoplast Preparation, Transfection and Dual-Luciferase Reporter Assay

The process of rice protoplast isolation was performed as described in the previous study with some modifications (Zhang et al., 2014). First, 10-day-old rice shoots were cut into approximately 0.5 mm strips and incubated in enzyme solution [1% cellulase R-10 (Yakult Honsha, Tokyo, Japan), 0.4% macerozyme R-10 (Yakult Honsha)] for 4 h in the dark with gentle shaking (80 rpm). After digestion, the pellets were washed with W5 solution, and the rice protoplasts were collected by centrifugation at 100 *g* for 10 min. Finally, 100 μl samples (5 × 10^5^–5 × 10^6^ cells) of the rice protoplasts were co-transfected with 10 μg plasmids of endogenous target mimics (eTMs), miRNAs, and luciferase, and the transfected protoplasts were incubated at 28°C to allow RNA expression. Each of the eTMs (MSTRG.66289.1 or MSTRG.52515.5) and the miRNA precursors (osa-miR156b and osa-miR396a) were individually cloned into the expression vector PCXUN under the control of the ubiquitin promoter, and the four target mRNA clones were separately inserted into the dual luciferase reporter vector pGreenII 0800-LUC (Hellens et al., 2005). The plasmids were introduced into rice protoplasts 20 h after transformation, and the luciferase activity was evaluated. The rice protoplasts were lysed using passive lysis buffer (Promega), and the luciferase activity was measured using a GloMax 20/20 Luminometer (Promega) and the Dual-Luciferase Reporter Assay System (Promega). The activity was normalized to *Renilla* luciferase activity. The experiments were performed in triplicate. The primer pairs used in gene cloning are listed in Supplementary Table 1.

## Results

### RNA Sequencing and Identification of lncRNAs in PTGMS Rice

**FIGURE 1:**
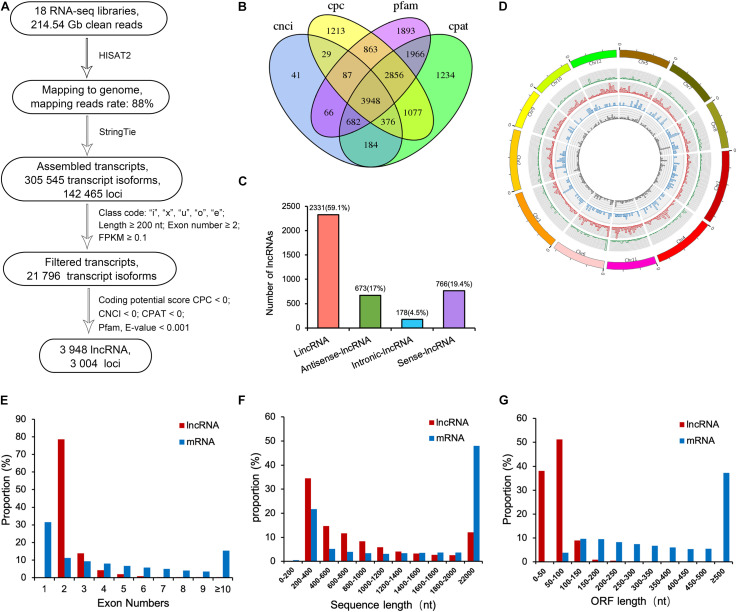
Computational pipeline for systematically identifying of lncRNAs in the PTGMS rice line WXS and their characteristics. **(A)** An integrative computational pipeline for the systematic identification of lncRNAs in WXS rice. **(B)** Venn diagram showing the numbers of potential lncRNAs filtered by four methods, including Pfam, CPC, CPAT, and CNCI. **(C)** Classification of identified lncRNAs. **(D)** Distribution of different types of lncRNAs. The concentric rings from outer to inner represent sense-lncRNAs (green), lincRNAs (red), antisense-lncRNAs (blue), and intronic-lncRNAs (gray), respectively, according to the loci of lncRNAs along each chromosome. **(E)** Distribution of exon numbers of lncRNAs and mRNAs. **(F)** Length distribution of lncRNAs and mRNAs. **(G)** Distribution of the open reading frame (ORF) lengths of lncRNAs and mRNAs.

To profile lncRNAs during the fertility transition in PTGMS rice, we performed ssRNA-seq in WXS-S and WXS-F during three different developmental stages, which were designated SP2, SP3, SP4, FP2, FP3, and FP4, respectively. Three biological replicates from each sample were analyzed, and 18 RNA-seq libraries were sequenced on an Illumina HiSeq Xten platform. We used a stringent filtering pipeline to identify lncRNAs from WXS rice (Figure 1A). After removing the adaptor sequences and low-quality reads from the raw reads, approximately 214.54 Gb clean reads were obtained, with an average of more than 10.23 Gb reads per sample. Moreover, we performed the Pearson’s Correlation Coefficient to detect the correlation of biological replications of RNA sequencing, and the result has confirmed the sample reproducibility (Supplementary Figure 1).

The clean reads of each sample were then sequence-aligned with the rice reference genome sequence (MSU_v7.0); the alignment efficiency ranged from 82.82% to 90.98% (Supplementary Table 2). The mapped sequences were then assembled, and a total of 305,545 transcripts corresponding to 142,465 loci were initially generated. Next, we selected the transcripts with class codes “I,” “x,” “u,” “o,” and “e,” removed transcripts that included a single exon, filtered out transcripts shorter than 200 nucleotides, and selected the transcripts with the FPKM ≥ 0.1. The remaining 21,796 transcripts were used for protein-coding capacity prediction by the CPC/CNCI/Pfam/CPAT four analytical methods (Figure 1B). Finally, a total of 3,948 reliably expressed lncRNAs corresponding to 3,004 loci were obtained (Supplementary Table 3).

### The Genomic Characteristics of lncRNAs in WXS

According to their locations in the genome, the 3,948 lncRNAs were divided into four types: 2,331 lincRNAs (59.1%), 673 antisense-lncRNAs (17%), 178 intronic-lncRNAs (4.5%), and 766 (19.4%) sense-lncRNAs (Figure 1C). Using the Circos program, these lncRNAs were mapped to the 12 chromosomes of the rice genome; we found that these lncRNAs were evenly distributed in all chromosomes with no obvious location preference (Figure 1D). In previous reports, lncRNAs have been found to contain fewer exons and to be shorter compared with mRNAs in plants (Zhang et al., 2014; Wang et al., 2017). Therefore, the exon numbers, transcript lengths and open reading frames (ORFs) of previously identified lncRNAs were compared with those of the 24,383 mRNAs produced by the RNA-seq in the present study. The exon number of the lncRNAs, as shown in Figure 1E, ranged from 2 to 13, and a majority of lncRNAs (78.6%) had two exons, much higher than the percentage of mRNAs that contained two exons. The average number of exons in lncRNAs was 2.4, less than the average of 8.4 exons found in mRNAs. The lengths of the majority of lncRNAs are shorter than those of mRNAs. For example, approximately 69.3% of lncRNAs ranged in size from 200 to 1,000 nt, and most of them were between 200 and 400 nt. In contrast, approximately 65.4% of the mRNAs were longer than 1,000 nt, and most were longer than 2,000 nt (Figure 1F). Moreover, 89.3% of lncRNA ORFs did not exceed 100 nt in length, while 96% of mRNA ORFs were longer than 100 nt (Figure 1G). In addition, the identified lncRNAs were aligned with the known lncRNAs in *Arabidopsis* that are listed in the NON-CODE database^3^ by BLASTN (*E* < 1e-5). Only 25 lncRNAs were found to be comparable with those known lncRNAs (Supplementary Table 3), indicating weak conservation of lncRNAs between *Arabidopsis* and rice WXS.

### Expression of lncRNAs at Three Pollen Developmental Stages

**FIGURE 2:**
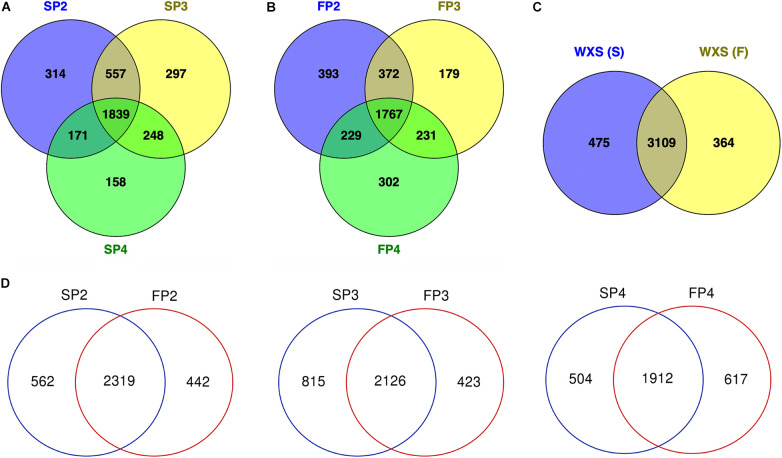
Venn diagram detailing shared and distinct lncRNAs expressed in the PTGMS rice line WXS at three pollen developmental stages. **(A)** Venn diagram showing the number of lncRNAs expressed at three different developmental stages of the male sterile line WXS-S. **(B)** Venn diagram showing the number of lncRNAs expressed at three different developmental stages of the male fertile line WXS-F. **(C)** Venn diagram showing the total number of lncRNAs expressed in the sterile and fertile lines. **(D)** Venn diagram showing the number of lncRNAs expressed at the three different developmental stages in WXS-S and WXS-F.

At three developmental stages in WXS-S, a total of 3,584 lncRNAs were obtained, of which 2,881, 2,941, and 2,416 were expressed at SP2, SP3, and SP4, respectively. Among them, 1,839 lncRNAs were expressed throughout the three stages, and that 314, 297, and 158 lncRNAs were specifically expressed at SP2, SP3, and SP4, respectively, (Figure 2A). In additions, a total of 3,473 lncRNAs were expressed in WXS-F, of which 2,761, 2,549, and 2,529 lncRNAs that were expressed at FP2, FP3 and FP4, respectively. Among them, 1,767 lncRNAs were expressed throughout the three stages, and that 393, 179, and 302 lncRNAs were expressed exclusively at FP2, FP3 and FP4, respectively (Figure 2B). The results showed that in both WXS-S and WXS-F, the stage P4 had the smallest number of total lncRNAs, the stage P2 had the largest number of specifically expressed lncRNAs.

We found that 3,109 lncRNAs were commonly expressed both in WXS-S and WXS-F, while 475 and 376 lncRNAs were specifically expressed in WXS-S and WXS-F, respectively (Figure 2C). At stages P2 and P3, the number of specifically expressed lncRNAs in WXS-S was greater than that in WXS-F (Figure 2D). In contrast, at the stage P4, fewer specifically expressed lncRNAs were found in WXS-S than in WXS-F.

### Analysis of Differentially Expressed lncRNAs

**FIGURE 3:**
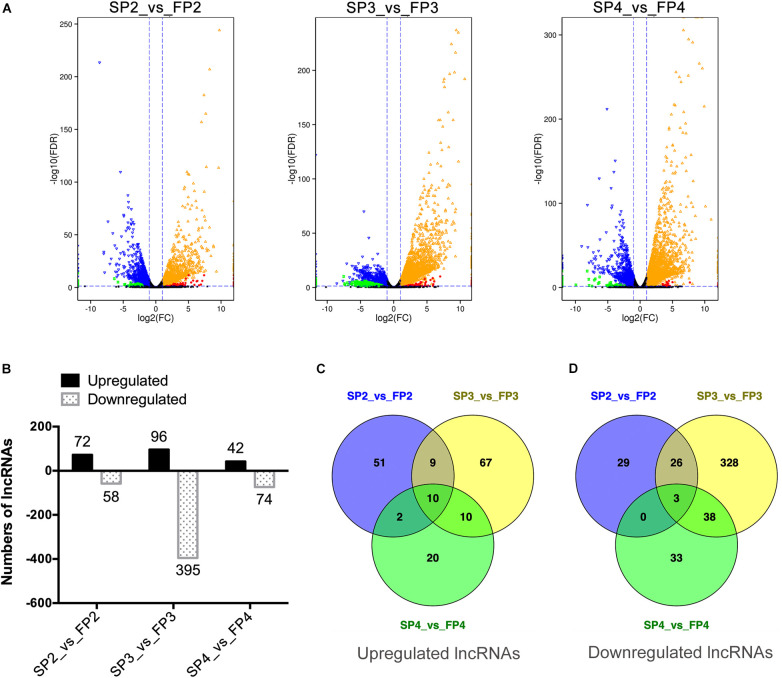
Differential expression of lncRNAs between WXS-S and WXS-F at three pollen developmental stages. **(A)** Volcano diagrams of differentially expressed genes (DEGs) and DE-lncRNAs in WXS-F compared with WXS-S at three pollen development stages. P-value < 0.05 and |log_2_(fold change)| ≥ 1 indicate that the differences in gene expression are significant. The red and green spots show DE-lncRNAs whose expression was up-regulated or down-regulated, respectively. The orange and blue spots show DEGs whose expression was up-regulated or down-regulated, respectively. The black spots indicate differences in gene expression that are not significant. **(B)** Number of up- and down-regulated DE-lncRNAs in WXS-F compared with WXS-S at three pollen developmental stages. **(C,D)** Venn diagrams showing the number of DE-lncRNAs that are commonly up- or down-regulated at three different developmental stages.

We found that thousands of genes, including genes encoding mRNAs and lncRNAs, were significantly differentially expressed [*P* < 0.05, |log_2_(fold change)| ≥ 1] between WXS-S and WXS-F while FPKM was used to estimate the expression level of each transcript (Figure 3A). In total, 7770 mRNAs were identified as DEGs, and 622 lncRNAs were identified as DE-lncRNAs (Supplementary Table 4). Among them, 130, 491, and 116 DE-lncRNAs were differentially expressed at stages P2, P3, and P4, respectively. Obviously, a large proportion of lncRNAs differentially expressed at the stage P3. As shown in Figure 3B, 72 lncRNAs were up-regulated, and 58 lncRNAs were down-regulated in FP2 compared to SP2. Although 96 lncRNAs were up-regulated in FP3 compared to SP3, the majority of lncRNAs (395) were down-regulated. Similarity, 42 lncRNAs were up-regulated, and 74 lncRNAs were down-regulated in FP4 compared to SP4. Moreover, 13 DE-lncRNAs showed similar trends throughout the three stages; ten of them were up-regulated in WXS-F, and three were down-regulated in WXS-F (Figures 3C,D). In particular, a large number of lncRNAs (328) was found to be specifically down-regulated in FP3 compared to SP3. These data suggest that lncRNAs whose expression profiles display significant differences may play a crucial role in the fertility transition of PTGMS rice.

To investigate the expression pattern of DE-lncRNAs in different developmental stages of WXS-S or WXS-F, we performed K-Mean Clustering and Hierarchical Clustering analysis using MeV software (Supplementary Figures 2, 3 and Supplementary Table 5). We obtained nine different clusters of the DE-lncRNAs in WXS-S and WXS-F, respectively. The developmental stage-specific expression pattern of lncRNAs suggested their specific roles in the development of rice panicle.

**FIGURE 4:**
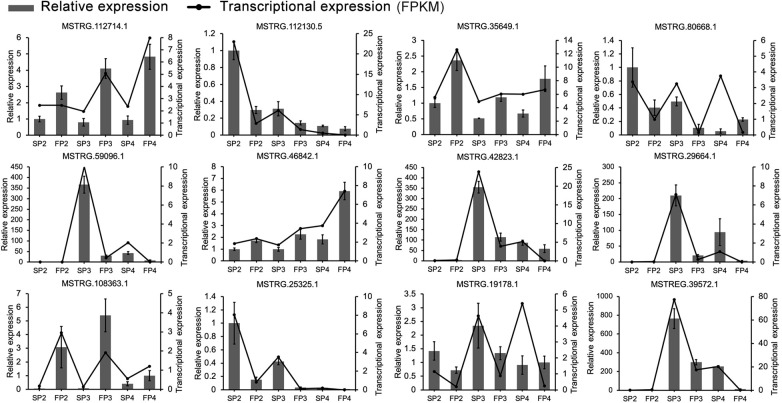
Validation of RNA-Seq data for the expression patterns of 12 randomly selected DE-lncRNAs using quantitative RT-PCR. The error bars indicate ± SD of three biological replicates. *OsActin1* was used as the internal reference.

To validate the RNA-seq data and examine the expression pattern of lncRNAs in WXS rice, we randomly selected 12 lncRNAs and quantified them at the three developmental stages of WXS using qRT-PCR (Figure 4). The results showed that the relative expression levels of lncRNAs in our experimental results were relatively consistent with the transcriptional expression levels determined by FPKM from RNA-seq data, indicating that our RNA-seq data had high repeatability and reliability.

### Prediction of Targets and Function of the DE-lnRNAs

It has been found that lncRNAs interact with adjacent and distant mRNA-encoding genes through *cis*- and *trans*-regulatory mechanisms. In the present study, we searched for potential *cis*-target genes within the regions 100 kb upstream and downstream of the identified lncRNAs as described by Huang et al. (2018). We also analyzed the complementary sequences present in all lncRNAs and mRNAs and used them to predict *trans*-target genes using LncTar software. As a result, 39,952 mRNA genes were found to be affected by *cis*-regulation of 3,948 lncRNAs, and 814 mRNA genes were found to be affected by *trans*-regulation of 354 lncRNAs. Among these, 622 DE-lncRNAs interacted with 9,746 *cis*-target genes, and four DE-lncRNAs interacted with seven *trans*-target genes (Supplementary Table 6). Obviously, *cis*-target genes represent an unexpectedly high fraction (99.9%) of the target genes of DE-lncRNAs.

**FIGURE 5:**
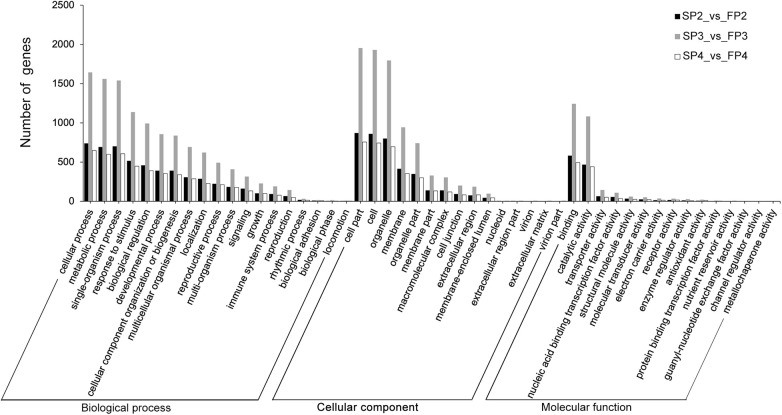
GO enrichment of the *cis*- and *trans*-target genes of DE-lncRNAs. The data shown on the left are sorted by number of target genes of DE-lncRNAs at P2, P3, and P4 stages, respectively. The black bars show the number of target genes expressed at the P2 stage; the gray bars show the number of target genes expressed at the P3 stage, and the white bars show the number of target genes expressed at the P4 stage.

To further investigate the potential role of DE-lncRNAs in the fertility transition, we performed functional annotations of the target genes of DE-lncRNAs (Supplementary Table 7). The GO enrichment analysis showed that the targets of DE-lncRNA in P2, P3, and P4 stages enriched in 46, 49, and 46 GO terms, respectively (Figure 5). Interestingly, three different stages were enriched in similar GO terms. In biological process, several important categories, including reproduction, reproductive process, growth, development process, metabolic process, biological regulation, response to stimulus, and signaling, were enriched. In cellular component, the three main categories were cell part, cell, and organelle; the membrane was also a represented category. In molecular function, most of the target genes were annotated as belonging to the categories of binding and catalytic activity, indicating that most of the DE-lncRNAs may play roles in binding and catalysis-associated functions. Our KEGG analysis of all target genes of DE-lncRNAs revealed a principal enrichment of these genes which related to metabolism adaptation, photosystem adaptation, hormone balance and transcriptional regulation (Supplementary Table 8).

### Identification of lncRNAs Related to Pollen and Anther Development in WXS Rice

To identify the lncRNAs that play important roles in pollen and anther development in the PTGMS rice line, we searched for DE-lncRNA target genes for which the terms “pollen development” (GO: 0009555) or “anther development” (GO: 0048653) appeared as functional annotations. In total, 150 target genes corresponding to 141 DE-lncRNAs were obtained in this way, and putative interactive networks for these genes were constructed using Cytoscape software. In these networks, one lncRNA may regulate one, two or multiple mRNA genes, and one mRNA gene may be regulated by multiple lncRNAs (Supplementary Figure 4).

**FIGURE 6:**
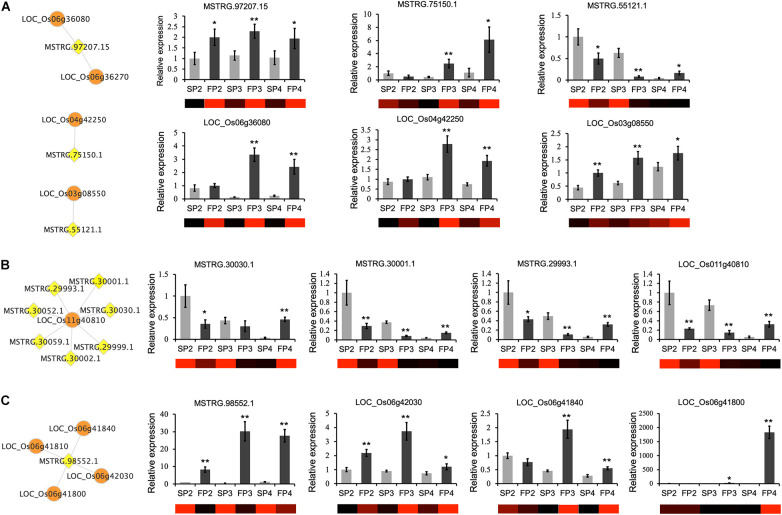
qRT-PCR confirmation of relative expression levels of lncRNAs and their target genes that play potential roles in the pollen and anther development. **(A)** The expression patterns of lncRNAs are similar or opposite to those of their corresponding target genes. **(B)** Multiple lncRNAs were predicted to regulate one target gene. The expression pattern of three lncRNAs and one target gene was verified by qRT-PCR. **(C)** One lncRNA was predicted to regulate several target genes. The expression pattern of one lncRNA and three corresponding target genes was verified by qRT-PCR. The networks show the predicted interaction between lncRNAs (yellow diamond nodes) and target mRNAs (orange round nodes). The heatmaps are generated from the FPKM values in the RNA-seq data. The means ± SDs of three biological replicates (*n* = 3) are shown. The asterisks indicate significant differences in WXS-F compared with WXS-S as determined by Student’s *t*-test (*P < 0.05; **P < 0.01).

To further understand the relationship between the DE-lncRNAs and their correlated target genes, we used qRT-PCR to measure the expression patterns of the DE-lncRNAs at three developmental stages in WXS-F and WXS-S. We found that the expression patterns of individual lncRNAs are similar or opposite to those of their corresponding target genes. For example, lncRNA MSTRG.97207.15 and its target gene, the gene encoding kinesin motor domain-containing protein (*LOC_Os06g36080*), were both up-regulated at all three developmental stages of WXS-F. MSTRG.75150.1 and its target gene, the gene encoding transferase family protein (*LOC_Os04g42250*), was up-regulated in both FP3 and FP4. MSTRG.55121.1 was down-regulated in WXS-F, but its target gene, the gene encoding STRUBBELIG-RECEPTOR FAMILY 6 precursor (*LOC_Os03g08550*), was up-regulated (Figure 6A). Moreover, a receptor kinase gene (*LOC_Os11g40810*) was predicted to be the target of seven lncRNAs, and our qRT-PCR results showed that the expression pattern of *LOC_Os11g40810* was similar to that of three lncRNAs (MSTRG.30030.1, MSTRG.30001.1, and MSTRG.29993.1) targeting it (Figure 6B). MSTRG.98552.1 has four potential target genes. The expression patterns of MSTRG.98552.1 and its three target genes were confirmed by qRT-PCR (Figure 6C).

### Prediction of the lncRNAs As Precursors of Known miRNAs in Rice

**FIGURE 7:**
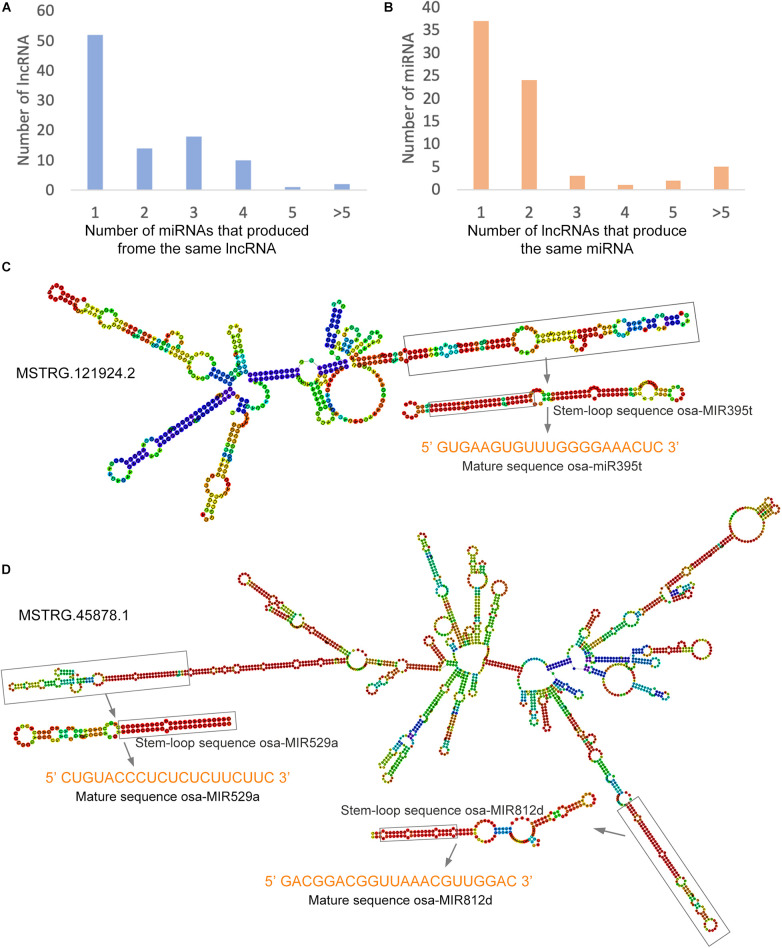
LncRNAs as precursors of miRNAs in the PTGMS rice line. **(A)** The number of miRNAs produced by lncRNAs. **(B)** Number of lncRNAs that produce the same miRNA. **(C)** Predicted secondary structure of MSTRG.121924.2, which contains only one precursor sequence of osa-miR395t. **(D)** Predicted secondary structure of MSTRG.121924.2, which contains two precursors of osa-miR529a and osa-miR812d. The predicted secondary structures were predicted using RNAfold. The color scale indicates high (red) to low (blue) probabilities of base pairing.

Studies have shown that lncRNAs act as precursors of small RNAs that regulate male sterility in rice (Ding et al., 2012a; Fan et al., 2016). In the present study, we aligned all identified lncRNAs with the rice miRNAs downloaded from miRbase and searched for potential miRNA precursors using the BLAST algorithm. A total of 97 lncRNAs were predicted as the precursors of 72 miRNAs (Supplementary Table 9). Among these, 53.6% of the lncRNAs may serve as precursors for only one miRNA, and the remaining lncRNAs may serve as precursors for two or more miRNAs (Figure 7A). In addition, 51.4% and 33.3% of the miRNAs might be produced from one or two lncRNAs, respectively, and a single miRNA might be produced from multiple different lncRNAs (Figure 7B). To explore the possible structural basis of the relationship between lncRNAs and miRNAs, we predicted the secondary structures of lncRNAs and pre-miRNAs using RNAfold^4^. For example, a long arm of the lncRNA MSTRG.121924.2 may be cleaved by an endonuclease and release the precursor sequence of osa-miR395t, ultimately forming mature osa-miR395t (Figure 7C). Moreover, MSTRG.45878.1 has two long arms that were predicted as precursor sequences of osa-miR529a and osa-miR812d (Figure 7D). These lncRNAs might participate in the fertility transition by regulating the expression of miRNAs.

### Prediction of lncRNAs As Endogenous Target Mimics of miRNAs in WXS Rice

**FIGURE 8:**
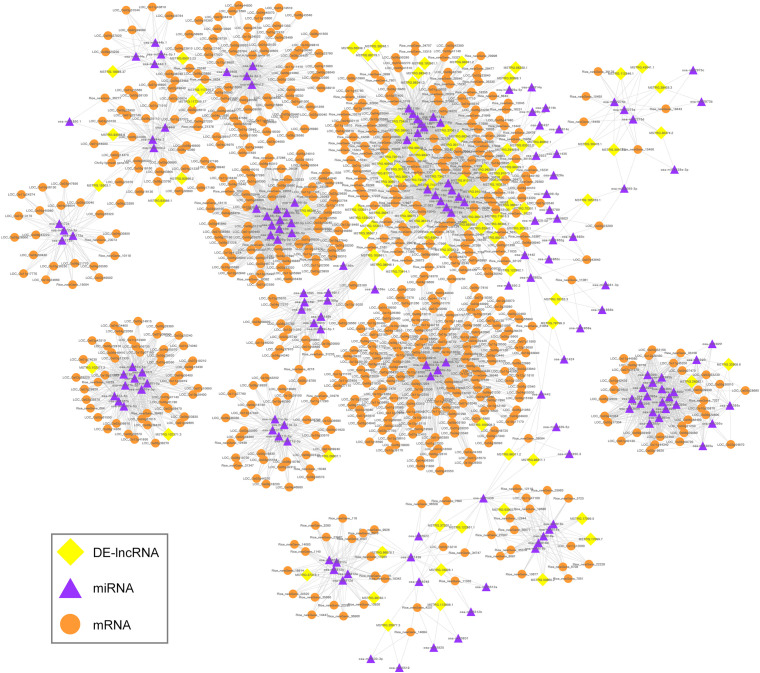
Global ceRNA networks in the PTGMS rice line. The yellow diamond-shaped, purple triangular, and orange round nodes represent DE-lncRNAs, miRNAs, and mRNAs, respectively.

Long non-coding RNAs have been reported to act as eTMs that interfere with interactions between miRNAs and their target mRNAs by binding to miRNAs through complementary sequences (Wu et al., 2013); these are also known as ceRNAs. In the present study, we predicted the lncRNA-mediated ceRNA networks that may occur during the fertility transition of WXS rice. In total, 246 lncRNAs were predicted to act as eTMs for miRNAs; the identified miRNAs included members of the well-known families osa-miR156, osa-miR160, osa-miR167, osa-miR169, osa-miR171, osa-miR172, osa-miR395, and osa-miR396 (Supplementary Table 10). Among these lncRNAs, 94 DE-lncRNAs are involved in the ceRNA interaction networks that were delineated by Cytoscape software. The networks contained 918 nodes and 5,061 edges; the nodes included 94 DE-lncRNAs, 150 miRNAs, and 674 mRNAs (Figure 8).

Using GO significant enrichment analysis with a mean *P*-value < 0.05 in the topGO R package, we found that the target mRNAs were significantly enriched for 859 GO terms, including a large number (681) of biological processes (Supplementary Table 11). The most highly enriched GO terms are highlighted in Supplementary Figure 5. The presence of 22 and 11 mRNAs that were significantly enriched in “regulation of flower development” (GO: 0009909) and “pollen tube growth” (GO: 0009860), respectively, suggests a role for lncRNAs in sexual reproduction. In addition, several target mRNAs were significantly enriched in terms related to floral development, including “pollen development” (GO: 0009555), “anther development” (GO: 0048653), and “floral organ formation” (GO: 0048449; Supplementary Table 11). Interestingly, six and 19 mRNAs were enriched in “response to high light intensity” (GO: 0009644) and “response to temperature stimulus” (GO: 0009266), respectively, indicating that lncRNAs may play roles in the response of rice to photoperiod and temperature and may thus be involved in the fertility transition of PTGMS rice.

### Experimental Validation of Two lncRNAs As eTMs for miRNAs in Rice Protoplasts

**FIGURE 9:**
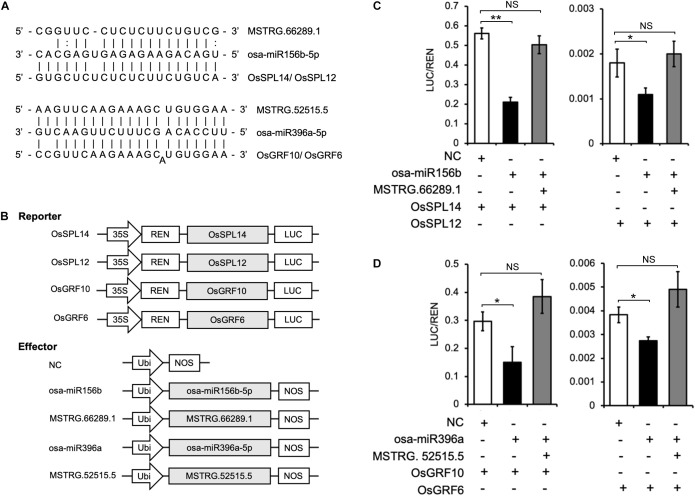
Dual luciferase reporter assay validation of two lncRNAs MSTRG.66289.1 and MSTRG.52515.5 acting as eTMs for osa-miRNA156 and osa-miRNA 396 to regulate SPL and GRF genes expression, respectively. **(A)** Predictive interaction sites among lncRNAs, miRNAs and the corresponding target genes. **(B)** Schematic diagram of the dual luciferase reporter and effector vector constructs. The coding sequences of *OsSPL14*, *OsSPL12*, *OsGRF10*, and *OsGRF6* were cloned into the reporter vector pGreenII 0800-LUC. The precursors of osa-miR156b-5p and osa-miR396a-5p, as well as the complete sequences of MSTRG.66289.1 and MSTRG.52515.5 were cloned into the effector vector pCXUN. LUC, firefly luciferase gene; REN, *Renilla* luciferase gene; and NC, negative control. **(C,D)** Dual luciferase reporter assay. The dual luciferase reporter vectors containing *OsSPL14*, *OsSPL12*, *OsGRF10*, or *OsGRF6* were co-transfected into rice protoplasts together with effector vectors containing the osa-miR156b-5p or osa-miR396a-5p precursors or with effector vectors containing the eTMs MSTRG.66289.1 or MSTRG.52515.5, as indicated in each assay. After 20 h, the luciferase activities were measured using the Dual Luciferase Reporter Gene Assay Kit. Error bars indicate the mean ± SD (*n* = 3). NS, no significant difference detected; *p < 0.05, **p < 0.01.

In the ceRNA networks prediction described above, the lncRNAs MSTRG.66289.1 and MSTRG.52515.5 were identified as potential eTMs for osa-miR156b-5p or osa-miR396a-5p, respectively (Figure 9A). Moreover, in agreement with previous studies, *OsSPL14* (*LOC_Os08g39890*) and *OsSPL12* (*LOC_Os06g4901*0) are targeted by osa-miR156, and *OsGRF10* (*LOC_Os02g45570*) and *OsGRF6* (*LOC_Os03g51970*) are targeted by osa-miR396 in rice (Xie et al., 2006; Liu et al., 2014). Therefore, based on the ceRNA hypothesis, MSTRG.66289.1 acts as an eTM of osa-miR156 to protect *OsSPL14* and *OsSPL12* from degradation; similarly, MSTRG.52515.5 can protect *OsGRF10* and *OsGRF6* from degradation by osa-miR396.

We further confirmed the predictive results by dual luciferase reporter assays using the dual luciferase reporter vector pGreenII 0800-LUC (Figure 9B). The results showed that the ratio of firefly fluorescence (LUC) to *Renilla* fluorescence (REN) was significantly decreased in rice protoplasts co-transfected with osa-miR156b and *OsSPL14* or *OsSPL12* compared with the negative control (Figure 9C). Interestingly, the LUC to REN ratio did not change significantly when the rice protoplasts were co-transfected with the vectors expressing osa-miR156b, MSTRG.66289.1, and *OsSPL14* or *OsSPL12*. Therefore, MSTRG.66289.1 might function as an eTM that inhibits osa-miR156b-5p and might regulate *OsSPL14* and *OsSPL12* expression indirectly. Similarity, overexpression of osa-miR396a significantly reduced the luciferase activity in rice protoplasts transformed with *OsGRF10* or *OsGRF6*, while its repressive effect was abrogated by the simultaneous overexpression of eTM MSTRG.52515.5 (Figure 9D). The results indicate that MSTRG.52515.5 acts as an eTM that interferes with the interaction between osa-miR396a-5p and its target genes *OsGRF10* and *OsGRF6*.

## Discussion

Male sterility is a particularly useful trait in hybrid seed production in plants and has contributed significantly to increased crop productivity worldwide. Rice PTGMS lines can regain their fertility under permissive environmental conditions, making them the major type of germplasm resource for the breeding of two-line systems (Chen and Liu, 2014; Fan and Zhang, 2017). The discovery of two rice lncRNAs, PMS1T and LDMAR, that regulate male sterility, suggested that lncRNAs play vital roles in PTGMS traits (Ding et al., 2012a; Fan et al., 2016). However, not all types of lncRNAs involved in the fertility transition of rice PTGMS lines have yet been identified and characterized at the genome-wide level. The recent transcriptome sequencing of multiple plant species has provided an unprecedented opportunity to elucidate the biological functions of plant lncRNAs. In the present study, we used ssRNA-seq to comprehensively screen lncRNAs and their target genes at three different developmental stages in WXS-S and WXS-F. The use of three biological replicates of libraries and of only high-quality data ensured that our ssRNA-seq data are extremely credible and reproducible, and this was validated by qRT-PCR. Our study will uncover the potential roles of lncRNAs in the pollen development and the fertility transition of PTGMS rice and is likely to provide valuable information that can be used in further functional analysis of lncRNAs.

### A Number of lncRNAs Are Differentially Expressed During the Fertility Transition of the PTGMS Rice Line WXS

Unlike previous studies that sought to identify abiotic stress-responsive lncRNAs, developmental process-related lncRNAs, and reproductive process-related lncRNAs in conventional rice (Johnson et al., 2018; Kiegle et al., 2018; Yuan et al., 2018), this study performed a genome-wide identification of lncRNAs and reliably characterized the lncRNAs involved in fertility transition in a PTGMS rice line. As a result, 3,948 lncRNAs were reliably identified. Previous studies have shown that rice lncRNAs are specifically and differentially expressed during reproduction. For instance, a number of lncRNAs were specifically expressed at a single developmental stage during sexual reproduction in rice (Zhang et al., 2014). An unexpectedly high fraction of most rice lincRNAs has been found to be specifically expressed in male gametes (Johnson et al., 2018). The phenotypic difference between WXS-S and WXS-F is only manifested in pollen development; in our study, the pollen was aborted due to the abnormal formation and abnormal meiosis of PMCs at the early pollen development stage in WXS-S (Zhang et al., 2016). Thus, using WXS as material enabled us to identify DE-lncRNAs in male-sterile and male-fertile lines and to trace the underlying fertility transition-related lncRNAs. In the present study, 622 lncRNAs were found to be significantly differentially expressed in WXS-S and WXS-F, and 12 of them were further verified by qRT-PCR analysis (Figure 4). The result indicated that lncRNAs might be involved in regulation of the fertility transition in the PTGMS rice line WXS. Moreover, a large proportion of lncRNAs differentially expressed at the stage of PMCs meiosis, suggested that this stage may be the most critical stage for fertility transition.

### Cis- and Trans-Regulation of Target Genes by lncRNAs Is Critical for the Fertility Transition of PTGMS Rice

It has been reported that lncRNAs regulate mRNA gene expression by *cis*- and *trans*-acting mechanisms based on their physical location and sequence complementarity, respectively (Ponting et al., 2009). In the present study, to further understand the functional implications of the identified lncRNAs, we predicted the *cis*- and *trans*-target genes of the lncRNAs and obtained functional annotations for the target genes of 622 DE-lncRNAs. The GO analysis showed that the targets of DE-lncRNAs play various roles in biological processes; some important GO terms related to the fertility transition, including reproduction, reproductive process, growth, development process, metabolic process, biological regulation, response to stimulus, and signaling, were obtained (Figure 5). The results are consistent with those of a study of TGMS-Co27 rice in which many genes that regulate temperature-inducible male sterility were found to be enriched in pollen development-related GO categories, including reproduction, developmental processes, responses to stimuli, and metabolic processes (Pan et al., 2014). In addition, it was reported that the target genes of the lincRNAs that are specifically expressed during the reproductive process were significantly enriched in reproduction-specific GO terms (Zhang et al., 2014). Therefore, the identified targets and the corresponding DE-lncRNAs may be involved in the fertility transition of PTGMS rice in our study. A core group of DE-lncRNAs whose target genes are involved in pollen and anther development according to the functional annotation were identified, and the expression patterns of several pairs of those lncRNAs and target genes were validated using qRT-PCR.

Furthermore, as shown in Supplementary Figure 6, distinct functional groups were found to be enriched among the target genes of DE-lncRNAs by KEGG pathway analysis; the identified functional pathways included metabolism adaptation, hormone balance, photosystem adaptation and transcription regulation. For example, *OsUgp2* (*LOC_Os02g02560*), a *cis*-target gene of MSTRG.40627.1, plays a role in carbohydrate metabolism, a subcategory of metabolism adaptation. It has been shown that *OsUgp2* is a pollen-preferential gene that is critical for starch accumulation and that its silencing leads to pollen sterility in rice (Mu et al., 2009). *CYP* (*LOC_Os01g11270*), a cytochrome P450 gene, is a target of three lncRNAs (MSTRG.2370.7, MSTRG.2370.9, and MSTRG.2385.2) and is related to lipid metabolism, another subcategory of metabolism adaptation. Two cytochrome P450 genes, *CYP703A3* and *CYP704B2*, were shown to be essential for the formation of anther cuticle and pollen exine during reproduction in male rice (Li et al., 2010; Yang et al., 2014). Furthermore, the lncRNAs MSTRG.6762.1 and MSTRG.6755.1 share the target gene *OsAIP1* (*LOC_Os01g36890*), which belongs to the functional group transcription regulation. *OsAIP1* is involved in the regulation of rice tapetum degeneration, and suppression of AIP1 results in pollen collapse and male sterility (Li et al., 2011). Moreover, several targets of DE-lncRNAs were found to participate in plant hormone signal transduction, including *OsbZIP08* (*LOC_Os01g59350*), *OsSAPK1* (*LOC_Os03g27280*), *OsSAUR4* (*LOC_Os02g05050*), *SLR1* (*LOC_Os03g 49990*), and *OsJAZ7* (*LOC_Os07g42370*). In addition, several of the identified target genes are involved in photosynthesis adaptation. The target of MSTRG.108363.1 and MSTRG.108363.2 is the gene encoding the photosynthesis-antenna protein *LHCII* (*LOC_Os07g38960*), which mainly functions in light capture and photoprotection in plants (Huang et al., 2013). Genes encoding proteins that function in photosynthesis and carbon fixation are important for starch synthesis (Jin et al., 2013), and we speculate that they might be essential for the response of PTGMS rice to photoperiod and temperature during anther development. Taken together, the identified lncRNAs might play roles in fertility transition by regulating both *cis-* and *trans*-target genes in our study. However, the functions of the identified lncRNAs need to be further experimentally confirmed in future studies.

### The Function of lncRNAs As Precursors and Target Mimics of miRNAs Is Essential for Fertility Transition of PTGMS Rice

Long non-coding RNAs have been reported to interact with miRNAs by acting as miRNA precursors, targets, or target mimics (Wilusz et al., 2009; Wu et al., 2013). Interestingly, the PGMS trait in NK58S is governed by two loci, *Pms1* and *Pms3*; both of these loci encode lncRNAs and produce small RNAs (Ding et al., 2012a; Fan et al., 2016), demonstrating the authenticity of lncRNAs acting as small RNA precursors to regulate male sterility. In the present study, we found that 97 lncRNAs could serve as precursors for 72 miRNAs. We also found that some lncRNAs produced more than one miRNA, although a large number of lncRNAs produced only one miRNA; for example, the lncRNA MSTRG.45878.1 contains the precursor sequences of *osa-miR529a* and *osa-miR812d*. It has been shown that *osa-miR529a* modulates rice panicle architecture by regulating the expression of the genes *OsSPL2*, *OsSPL14*, and *OsSPL17* (Yue et al., 2017). The *osa-miR812* family members showed significant different expression between WXS-S and WXS-F in our previous study (Zhang et al., 2016).

Recently, several lncRNAs in rice and *Arabidopsis*, including osa-eTM160-3, ath-eTM160-1, and ath-eTM166-1, have been demonstrated to be functional eTMs (or ceRNAs) involved in the regulation of plant development (Wu et al., 2013). Moreover, two lncRNAs in *Brassica rapa*, bra-eTM160-1 and bra-eTM160-2, were experimentally verified to be eTMs for bra-miR160-5p and to function in pollen development and male fertility (Huang et al., 2018). In the present study, a total of 94 DE-lncRNAs were identified as potential eTMs for 150 miRNAs, and the ceRNA interaction networks that were construct. In addition, as show in Supplementary Figure 6, three functional groups including metabolism adaptation, hormone balance, and transcription regulation, were obtained by KEGG analysis of the target mRNAs. Previous studies have confirmed that some transcription factors (TFs) are key regulators of male reproductive development in rice. For example, *GAMYB* is an important component of GA signaling and has an important role in rice anther development (Aya et al., 2009), and *MADS* has been shown to regulate the anther development and pollen maturation (Hu et al., 2011; Liu et al., 2013). Interestingly, in the study we also found that many target mRNAs encode TFs that are involved in transcriptional regulation. For examples, the lncRNA MSTRG.65013.23 was predicted to compete with *MYB* (*LOC_Os01g09640*) for osa-miR444a, and four lncRNAs (MSTRG.18898.37, MSTRG.65013.23, MSTRG.117256.2, and MSTRG.117259.17) were predicted targets of osa-miR444a, which competes with *MADS* TF family. Above all, our results imply that lncRNAs might participate in ceRNA networks and thereby help regulate the fertility transition of rice PTGMS.

In the present study, using dual luciferase reporter assays in WXS rice protoplasts demonstrated that MSTRG.66289.1 and MSTRG.52515.5, as eTMs of osa-miR156b and osa-miR396a, regulated the expression of the *SPL* and *GRF* genes, respectively. Previous studies have confirmed that the osa-miR156 target gene *SPL* functions in various developmental processes, especially flower development in rice (Xie et al., 2006). *OsSPL14*, also known as *IPA1/WFP*, is regulated by osa-miR156, and a point mutation at the target site leads to increased expression (Jiao et al., 2010; Zhang et al., 2017). Besides, osa-miR396 and its targets, which are *GRF* TFs, have been reported as potential regulators of flowering time, floral organogenesis, grain shape, panicle length and seed shattering in rice (Liu et al., 2014; Sun et al., 2016). Here, using overexpression of the lncRNA MSTRG.66289.1 to perturb osa-miR156-directed regulation of *OsSPL14* and *OsSPL12*, we found that the repressive effect of miR156b was decreased and the expression level of mRNAs were increased. Similarly, the effect that osa-miR396a-directed regulation of *OsGRF10* and *OsGRF6*, was abrogated when transient transformation and overexpression of the predicted eTM MSTRG.52515.5 occurred simultaneously. Taken together, the lncRNAs MSTRG.66289.1 and MSTRG.52515.5 act as eTMs and are promising candidates involved in fertility transition in the PTGMS rice line. This hypothesis requires further experimental investigation. Our results provide specific clues that can be used as a starting point to fully elucidate the molecular mechanisms of fertility transition in the PTGMS rice line.

## Data Availability Statement

The sequencing data have been deposited in the Sequence Read Archive (SRA) at the National Center for Biotechnology Information (NCBI) under the accession number GSE125608.

## Author Contributions

YD, YW, and HZ conceived and designed the experiments. YW and QL performed the experiments. YW, JJ, HC, YZ, and QL gathered samples. XH supported the materials. YW participated in the analysis of the data and drafted the manuscript. YD revised the manuscript. All authors have read and approved the final version of the manuscript.

## Conflict of Interest

XH was employed by the company Wuhan Wuda Tianyuau Bio-Tech Co., Ltd. The remaining authors declare that the research was conducted in the absence of any commercial or financial relationships that could be construed as a potential conflict of interest.

## Footnotes

1 http://rice.plantbiology.msu.edu/pub/data/Eukaryotic_Projects/o_sativa/annotation_dbs/pseudomolecules/version_7.0/

2 http://www.mirbase.org

3 http://www.non-code.org

4 http://rna.tbi.univie.ac.at/cgi-bin/RNAWebSuite/RNAfold.cgi

## Supplementary Material

Supplementary Figure 1 The diagram of the correlation of biological replications using the Pearson’s Correlation Coefficient.

Supplementary Figure 2 Genome-wide gene expression profiles of all DE-lncRNAs in WXS-S rice. **(A)** The Hierarchical clustering of the DE-lncRNAs in different developmental stages of WXS-S. **(B)** Heat map of DE-lncRNAs by the K-means clustering method.

Supplementary Figure 3 Genome-wide gene expression profiles of all DE-lncRNAs in WXS-F rice. **(A)** The Hierarchical clustering of the DE-lncRNAs in different developmental stages of WXS-F. **(B)** Heat map of DE-lncRNAs by the K-means clustering method.

Supplementary Figure 4 Interaction networks between lncRNAs and mRNAs in the PTGMS rice line.

Supplementary Figure 5 GO significantly enrichment for target mRNAs in the ceRNA networks.

Supplementary Figure 6 Possible regulatory mechanism of DE-lncRNAs involved in the fertility transition.

Supplementary Table 1 Primer list for gene-specifi primers.

Supplementary Table 2 RNA-seq data statistics.

Supplementary Table 3 Characteristics of 3948 identified lncRNAs expressed in the PTGMS rice line WXS.

Supplementary Table 4 Statistics of significantly differentially expressed lncRNAs and mRNAs.

Supplementary Table 5 Statistics of the expression profiles of DE-lncRNAs by K-Mean Clustering and Hierarchical Clustering analysis.

Supplementary Table 6 The *cis*- and *trans*-target genes of the identified lncRNAs.

Supplementary Table 7 Functional annotations of the target genes of DE-lncRNAs in WXS rice.

Supplementary Table 8 KEGG pathway analysis of all target genes of DE-lncRNAs in WXS rice.

Supplementary Table 9 The predicted lncRNAs function as miRNA precursors in rice.

Supplementary Table 10 The predicted lncRNAs function as endogenous target mimics of miRNAs in WXS rice.

Supplementary Table 11 TopGO significant enrichment analysis of the target mRNAs in the ceRNA networks.
