# Supplementary material for: Genome-Wide Identification of lncRNAs Involved in Fertility Transition in the Photo-Thermosensitive Genic Male Sterile Rice Line Wuxiang S
Source: Front Plant Sci. 2021 Jan 14;11:580050. doi: 10.3389/fpls.2020.580050 (PMC7840536; doi:10.3389/fpls.2020.580050)
Supplement: Supplementary Figure 1 — The diagram of the correlation of biological replications using the Pearson’s Correlation Coefficient. [file Data_Sheet_1.PDF]

Supplementary material

Figure S1

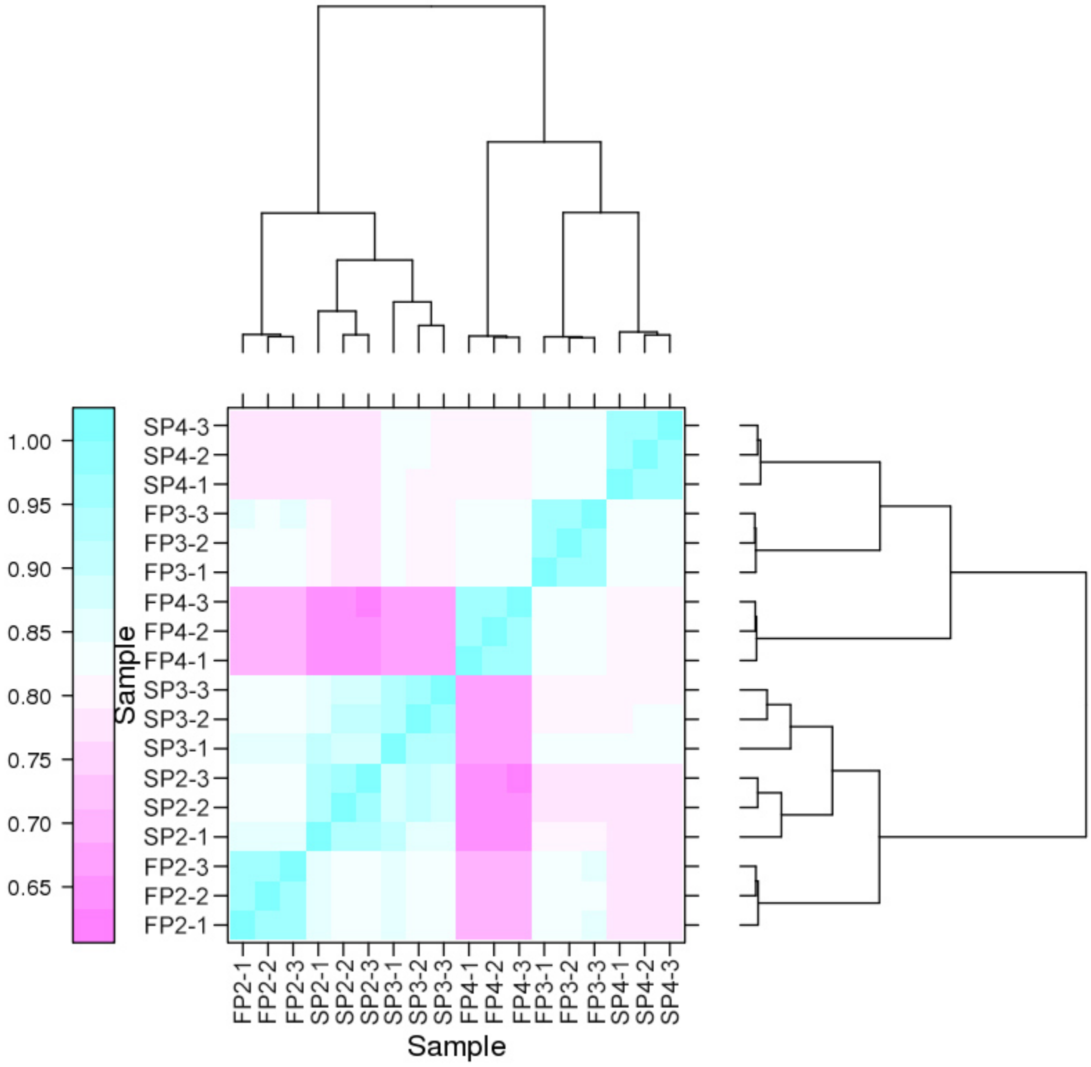

Supplementary Figure S1. The diagram of the correlation of biological replications using the Pearson's Correlation Coefficient. The abscissa and ordinate in the figure are sample numbers, and their order is determined by the cluster results of the sample correlation. The top and right sides of the graph are corresponding clustering trees. The color of graph reflects the correlation between biological replications, and the corresponding relationship is shown in the legend on the left.

Figure S2

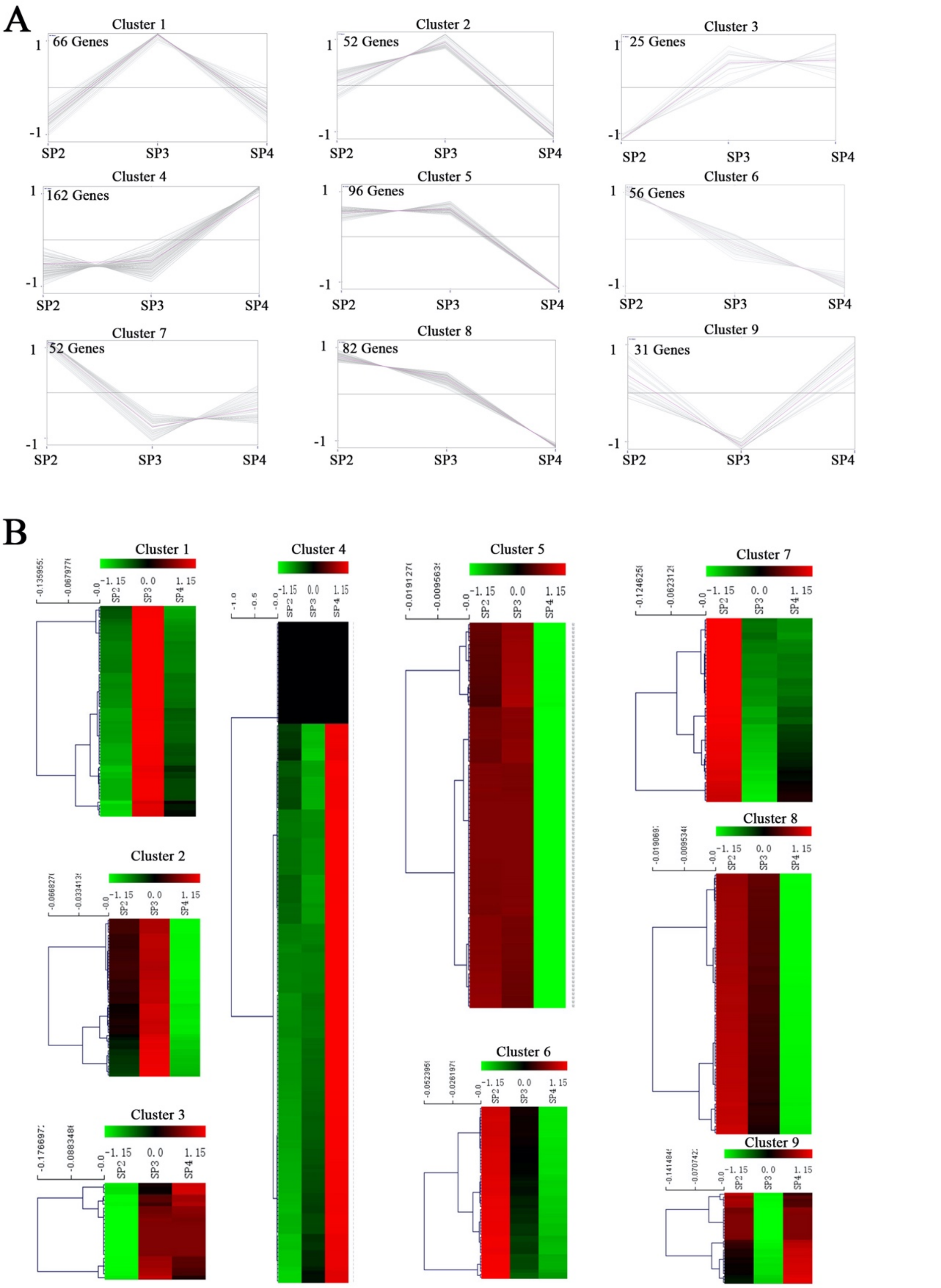

Supplementary Figure S2. Genome-wide gene expression profiles of all DE-lncRNAs in WXS-S rice. (A) The Hierarchical clustering of the DE-lncRNAs in different developmental stages of WXS-S. (B) Heat map of DE-lncRNAs in WXS-S by the K-means clustering method.

Figure S3

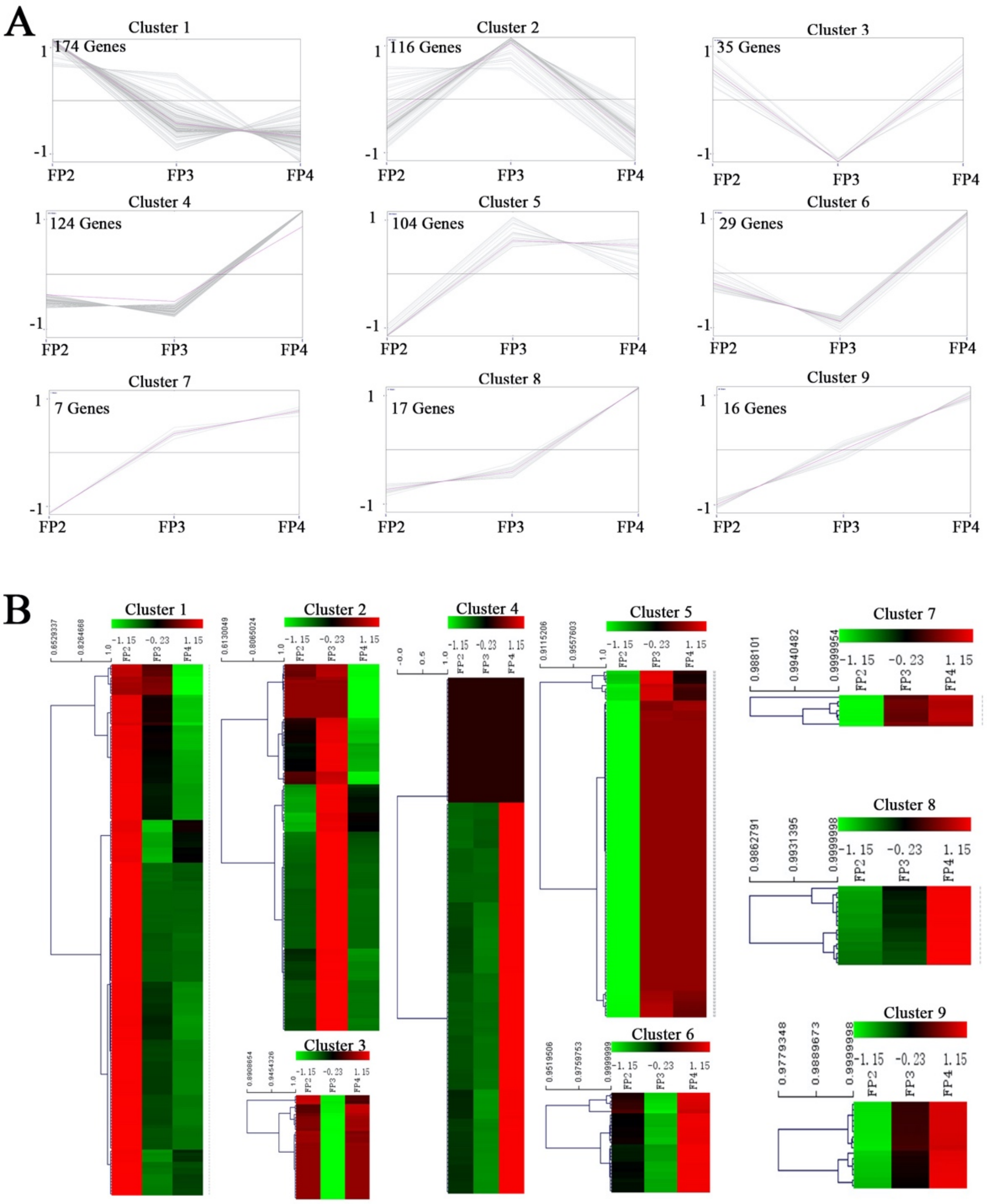

Supplementary Figure S3. Genome-wide gene expression profiles of all DE-lncRNAs in WXS-F rice. (A) The Hierarchical clustering of the DE-lncRNAs in different developmental stages of WXS-F. (B) Heat map of DE-lncRNAs by the K-means clustering method.

Figure S4

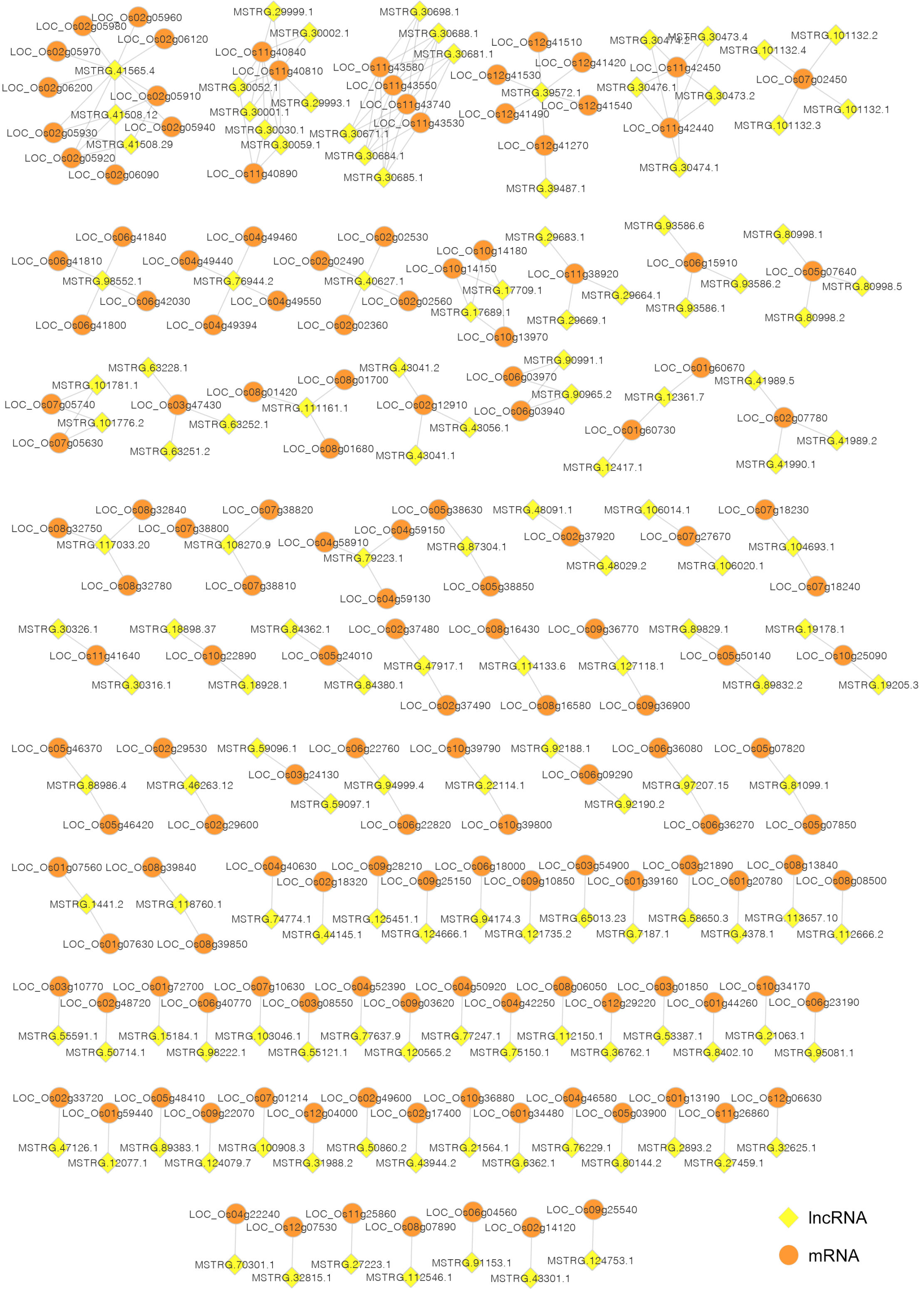

Supplementary Figure S4. Interaction networks between lncRNAs and mRNAs in the PTGMS rice line. The diamond and round nodes represent lncRNAs and mRNAs, respectively.

Figure S5

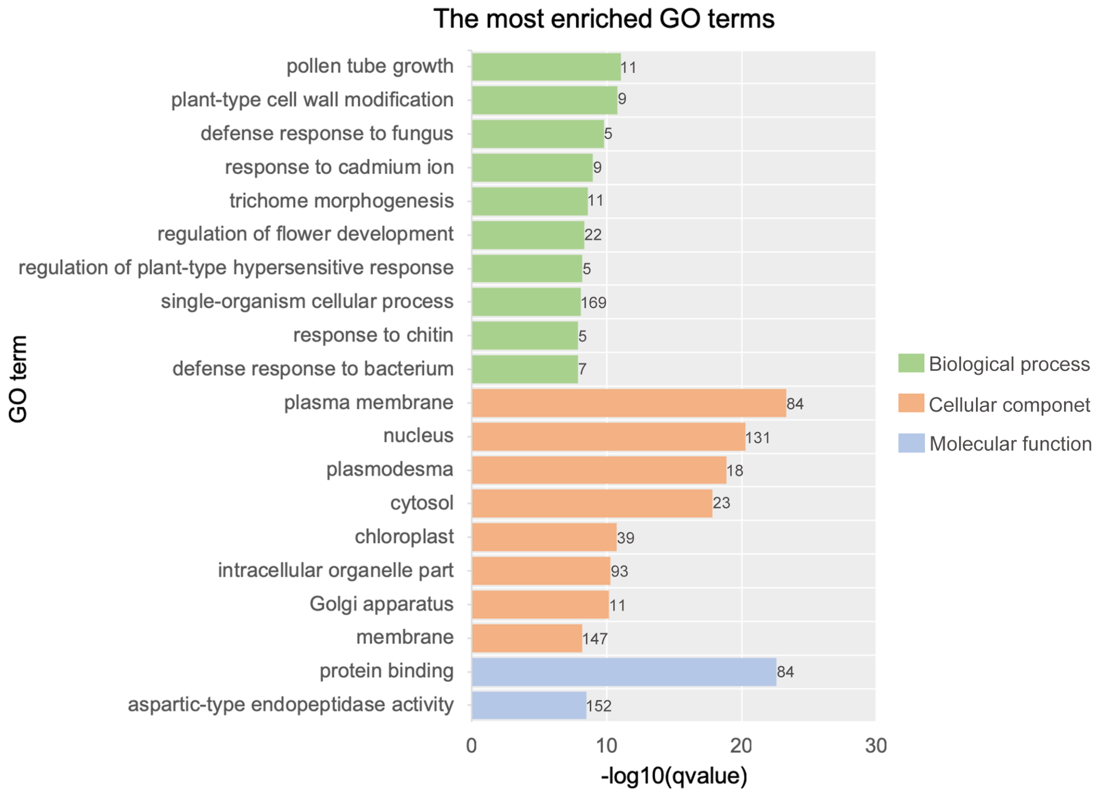

Supplementary Figure S5. GO significantly enrichment for target mRNAs in the ceRNA networks. The reliability is calculated by -$\log_{10}$(q-value). This figure just displays 20 the most enriched GO terms and more detailed information is given in Supplementary Tables S9.

Figure S6

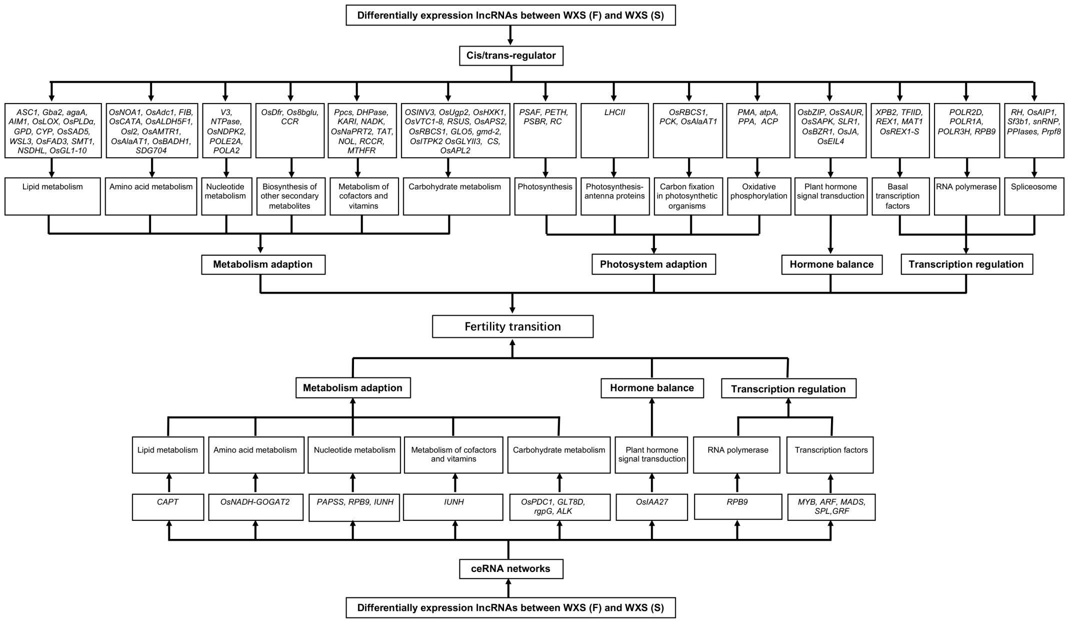

Supplementary Figure S6. Possible regulatory mechanism of DE-lncRNAs involved in fertility transition. Function prediction suggested that the identified lncRNAs participate in regulation of the fertility transition by regulating *cis*- and *trans*-target genes and by acting as miRNA precursors as well as eTMs for miRNAs. The functional lncRNA candidates may play pivotal roles in modulating metabolism adaptation, photosynthesis, hormone balance, and transcription regulation.
